# Is proximal gastrectomy indicated for locally advanced cancer in the upper third of the stomach?

**DOI:** 10.1002/ags3.12486

**Published:** 2021-07-16

**Authors:** Motonari Ri, Koshi Kumagai, Ken Namikawa, Shinichiro Atsumi, Masaru Hayami, Rie Makuuchi, Satoshi Ida, Manabu Ohashi, Takeshi Sano, Souya Nunobe

**Affiliations:** ^1^ Department of Gastroenterological Surgery Cancer Institute Hospital Japanese Foundation for Cancer Research Tokyo Japan; ^2^ Department of Gastroenterology Cancer Institute Hospital Japanese Foundation for Cancer Research Tokyo Japan

**Keywords:** distal margin, locally advanced gastric cancer, lymph node metastasis, proximal gastrectomy, therapeutic index, upper third gastric cancer

## Abstract

**Aim:**

To treat upper third gastric cancer, proximal gastrectomy (PG), a function‐preserving procedure, is recommended for early lesions when at least half the distal stomach can be preserved, while total gastrectomy (TG) is standard for locally advanced lesions. Oncological feasibility, when applying PG for such lesions, remains unknown.

**Methods:**

We reviewed patients undergoing TG for clinical (c) T2–T4 upper third gastric cancer between 2006 and 2015. Preoperative tumor locations were further classified into the cardia, fornix, and gastric body based on endoscopic findings. The metastatic rate and therapeutic value index for lymph node (LN) dissection were determined, and characteristics of patients with distal LN (No. 4d, 5, and 6) metastasis (DLNM) were reviewed. In addition, patients with pathological tumor invasion to the middle third (M) region were investigated.

**Results:**

We studied 167 patients. There were 8 (4.8%) with DLNM and 41 (24.6%) with pathological tumor invasion to the M region. As to regional stations, therapeutic indices for LN dissection at stations No. 4d, 5, 6, and 12a were zero or extremely low. No DLNM was detected in cT2 lesions or cT3/T4 lesions located within the cardia and/or the fornix. In addition, none of the lesions located within the cardia and/or the fornix by preoperative endoscopy extended to the M region in the pathological specimen.

**Conclusions:**

For upper third gastric cancer, PG without No. 12a dissection might be acceptable for cT2–T4 lesions located within the cardia and/or the fornix when considering the risk of DLNM and cancer‐positivity in the distal stump.

## Graphical abstract

The metastatic rate and therapeutic value index for distal lymph node (No. 4d, 5, and 6) were investigated in patients undergoing total gastrectomy for cT2–T4 upper third gastric cancer. In addition, pre‐ and postoperative tumor location was reviewed by preoperative endoscopy and the pathological specimen. For upper third gastric cancer, proximal gastrectomy might be acceptable for cT2–T4 lesions located within the cardia and/or the fornix when considering the risk of distal lymph node metastasis and cancer‐positivity in the distal stump.

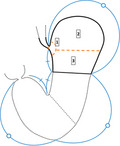

## 1 INTRODUCTION

Worldwide, gastric cancer is among the most life‐threatening malignant neoplasms.^1^, ^2^ The incidence of gastric cancer in the upper third of the stomach has recently been rising in both Western and Asian countries.^3^, ^4^, ^5^ As a therapeutic strategy for upper third gastric cancer indicated for surgical treatment, proximal gastrectomy (PG), a function‐preserving procedure, is advocated for lesions diagnosed at an early stage when more than half of the distal stomach can be preserved.^6^ In contrast, total gastrectomy (TG) is now the standard procedure for locally advanced lesions in the upper third of the stomach.^6^

Comparing TG and PG for early gastric cancer, PG is considered to be more advantageous in mitigating body weight loss, maintaining nutritional status, and not causing deterioration of quality of life postoperatively.^7^, ^8^, ^9^ Therefore, provided that oncological safety is assured, PG may also be the preferred surgical treatment for locally advanced gastric cancer in the upper third region. As to esophago‐gastric junctional (EGJ) cancer, PG can be selected even for an advanced tumor if the primary lesion is less than 4 cm in size, based on the oncological safety of lymph node (LN) metastasis, as stated in the Japanese gastric cancer treatment guidelines.^6^

However, there are oncological concerns when applying PG for advanced upper third gastric malignancies other than EGJ tumors. That is, an optimal extent of LN dissection in performing PG for advanced cancer has not yet been established, with only a few reports focusing on this issue,^10^, ^11^ nor has the relationship between actual locations of primary lesions and LN metastasis been investigated in sufficient detail. Moreover, it is essential to ensure an adequate distal tumor margin and sufficient volume of the remnant stomach after gastric dissection.

Here we evaluated pathological metastasis involving the aforementioned regional LNs and the distal tumor margin in patients undergoing TG for clinically advanced gastric cancer in the upper third of the stomach. The present results are anticipated to contribute to determining the criteria for applying PG to advanced lesions.

## 2 METHODS

### 2.1 Patients

From January 2006 to December 2015, patients who underwent TG for cT2–4 gastric cancer, preoperatively diagnosed as being located within the upper third region of the stomach without or with esophageal invasion, at the Department of Gastroenterological Surgery, Cancer Institute Hospital, Tokyo, Japan, were registered in this study. Although D2 LN dissection was usually performed during TG for advanced lesions, a few patients underwent TG with D1/D1+ LN dissection at the discretion of the main surgeon, based on patient background factors such as age, performance status, comorbidities, and so on. The exclusion criteria were as follows: (a) EGJ cancer with its center located within 2 cm of the EGJ, as defined by the Japanese gastric cancer treatment guidelines,^12^ (b) macroscopic type 4 (diffuse infiltrative) lesion, (c) remnant gastric cancer, (d) simultaneous multiple gastric cancers, (e) the presence of other primary malignant disease, (f) history of preoperative chemotherapy, and (g) R1/R2 resection. This study was approved by the Institutional Review Board of the Cancer Institute Hospital (No. 2017‐1187).

### 2.2 Assessment of clinical staging

For clinical T factor diagnosis, findings obtained by endoscopy and the features noted on computed tomography (CT) by an experienced radiologist were reviewed, and the depth of tumor invasion of the wall was finally determined at the gastric cancer team conference including surgeons, endoscopists, and chemotherapists. Regional LNs with a long‐axis diameter of 10 mm or more on CT were diagnosed as metastatic nodes and their station numbers were also examined. Clinical stages were determined according to the 14th edition of the Japanese Classification of Gastric Carcinoma.^13^

### 2.3 Pathological metastasis and therapeutic value index for LNs at each station

As to the regional LNs to be dissected, the rate of LN metastasis and the therapeutic value index for dissection of LNs at each station were examined, including LNs not defined as meeting the extent of lymphadenectomy in PG for early cancer. The rate of metastasis was calculated by dividing the number of patients with metastasis at the nodal station by the number of patients in whom that station was retrieved. Moreover, the therapeutic values of each dissected LN were determined using the therapeutic value index proposed by Sasako et al.^14^ This index was obtained by multiplying the rate of nodal metastasis by the 5‐y overall survival (OS) for each nodal station. The 5‐y OS in patients with LN metastasis was calculated for each station, irrespective of nodal metastasis at other stations. In addition, clinical details of patients with distal LN metastasis (DLNM), that is, metastasis to LNs at stations No. 4d, 5, and/or 6, which could not be removed during PG, were investigated.

### 2.4 Evaluation of tumor location based on preoperative endoscopy and pathological specimen

**FIGURE 1:**
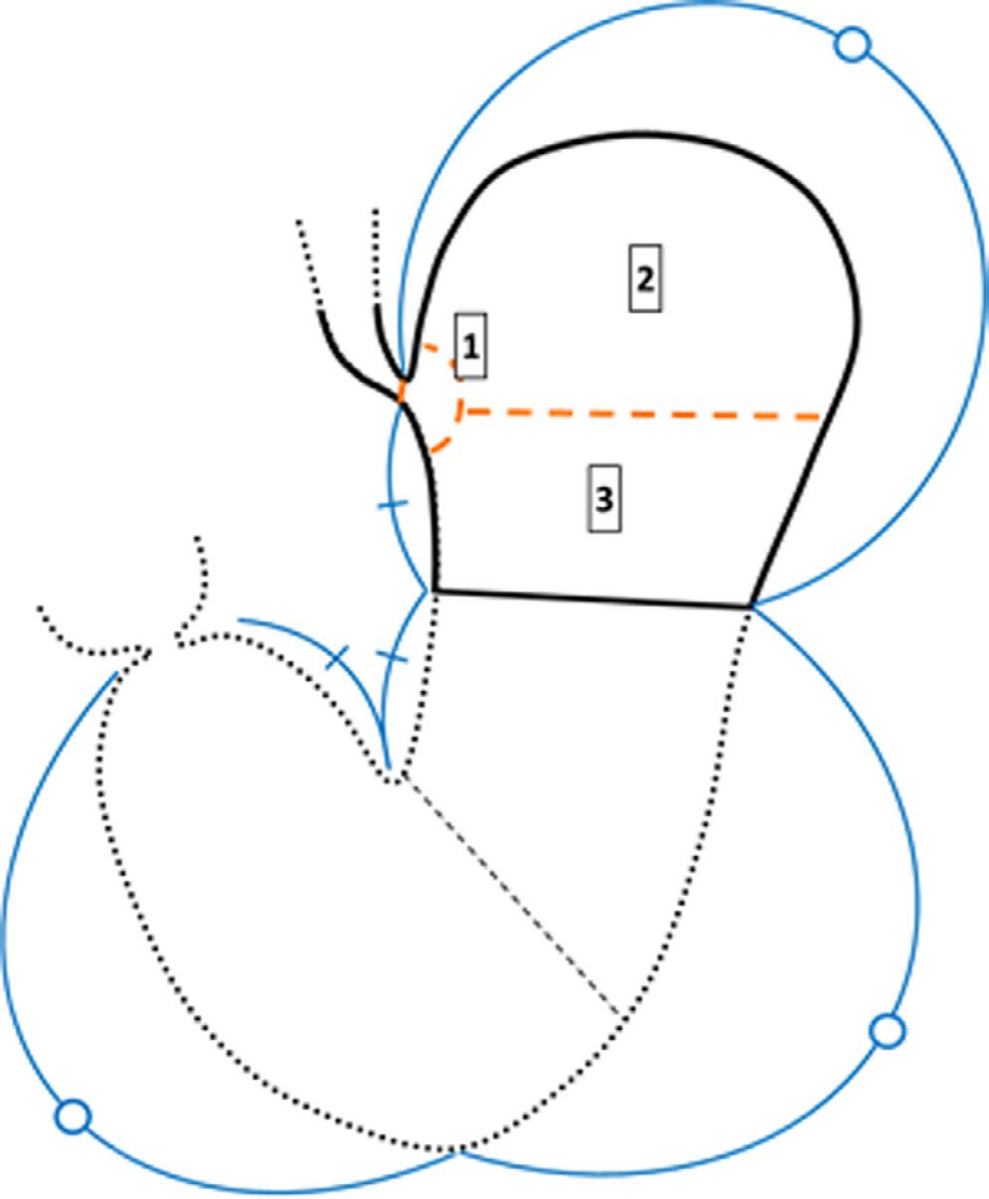
Classification of tumor location in the upper third of the stomach based on endoscopic findings. Locations of the distal tumor border in gastric cancers of the upper third were divided into three regions based on the endoscopic findings: (1) the cardia, (2) the fornix, (3) the gastric body

**FIGURE 2:**
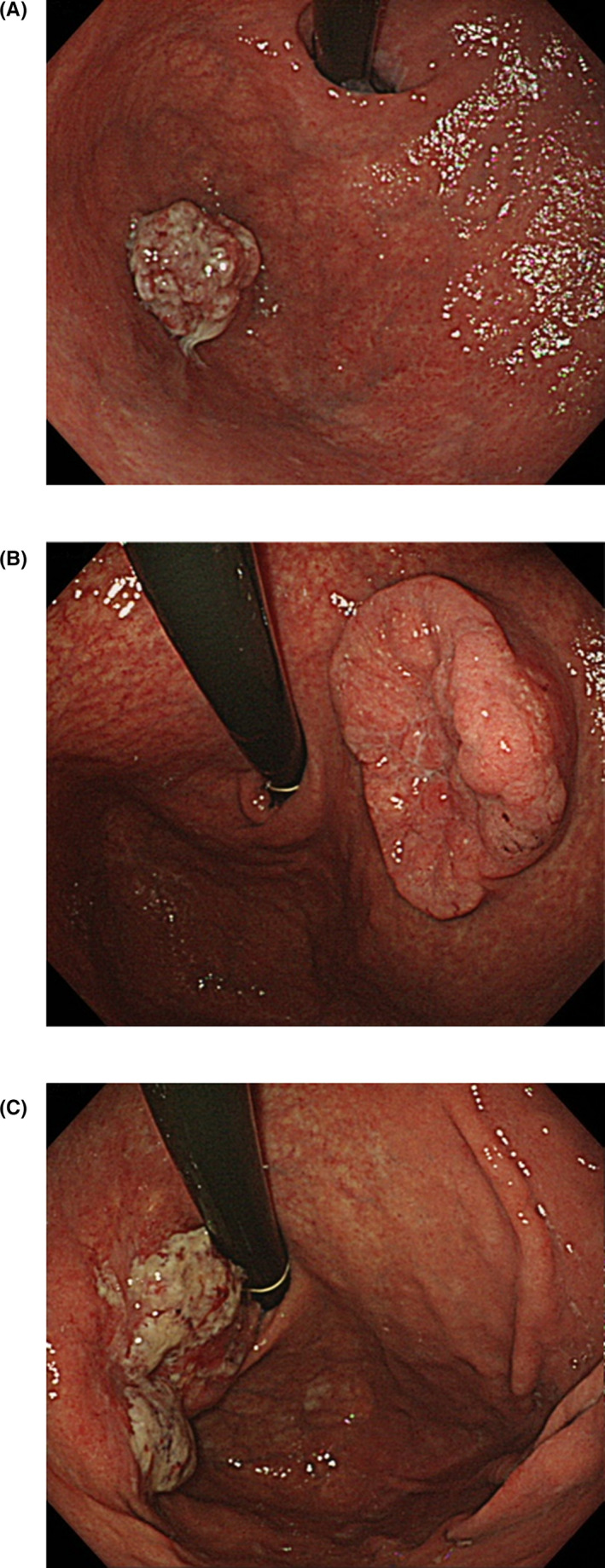
Representative endoscopic photographs showing tumor locations. (A) Located within the fornix. (B) Located within the gastric body. (C) Located in both the gastric body and the cardia

We assessed tumor location preoperatively based on the endoscopic findings. In particular, the locations of the distal tumor border were further divided into three regions, ie, the cardia, the fornix, and the gastric body (Figure 1). The location of the cardia was defined as being within 2 cm of the EGJ in the stomach. Representative endoscopic photographs of tumors included in this study are presented in Figure 2. In addition, tumor locations were also determined postoperatively using the pathological specimen, and divided into the upper third or middle third (M) regions of the stomach.

## 3 STATISTICAL ANALYSIS

The patient background characteristics, surgical details, and pathological findings were collected from our database and information contained in electronic medical records. The relationships between clinical characteristics and pathological findings, including LN metastasis and tumor location, were investigated. All continuous variables are expressed as median values. Statistical analyses were conducted using the Mann–Whitney *U*‐test and the chi‐squared test. A *P*‐value less than .05 was considered to indicate a statistically significant difference. All statistical analyses were performed with JMP Pro 13 (SAS Institute Japan, Japan) for windows.

## 4 RESULTS

### 4.1 Clinicopathological characteristics

Patient clinical characteristics are shown in Table 1. In total, 167 patients were included in this study. As to the location of the distal tumor border according to preoperative endoscopy, 12 patients (7.2%) had lesions limited to the cardia or the fornix. Although 23 patients (13.8%) were clinically diagnosed as having LN metastasis, none of them had swollen LNs at stations No. 4d, 5, and/or 6. As to pathological findings (Table 2), there were 8 patients (4.8%) with DLNM, and 41 (24.6%) showing tumor invasion to the M region in the pathological specimen.

**TABLE 1:** Characteristics of the patients

	All (n = 167)
Sex, n (%)	
Male	116 (69.5)
Female	51 (30.5)
Age, years [IQR]	67 [59‐73]
Tumor location, n (%)	
U	151 (90.4)
UE	16 (9.6)
Location of distal tumor border by endoscopy, n (%)	
Cardia or fornix	12 (7.2)
Gastric body	155 (92.8)
Tumor circumference, n (%)	
Less	46 (27.5)
Gre	22 (13.2)
Ant	24 (14.4)
Post	71 (42.5)
Circ	4 (2.4)
Clinical macroscopic type, n (%)	
0	35 (21.0)
1	14 (8.4)
2	39 (23.4)
3	76 (45.5)
5	3 (1.8)
Preoperative tumor size, mm [IQR]	40 [30‐55]
Clinical T factor, n (%)	
T2	65 (38.9)
T3	41 (24.6)
T4	61 (36.5)
Clinical N factor, n (%)	
N0	144 (86.2)
N+	23 (13.8)
Region of clinical lymph node metastasis	
Left area of cardia (No. 2)	1 (4.2)
Lesser curvature (No. 1 and 3)	21 (87.5)
Supra‐pancreas (No. 7, 8a, 9 and 11p)	2 (8.3)
Splenic hilum (No. 10)	0 (0)
Distal area (No. 4d, 5 and 6)	0 (0)
Surgical approach, n (%)	
Open	163 (97.6)
Laparoscopic	4 (2.4)
Type of lymph node dissection, n (%)	
D2 or more	156 (93.4)
D1 or D1+	11 (6.6)

Abbreviation: IQR, interquartile range.

**TABLE 2:** Pathological findings

	All (n = 167)
Pathological tumor size, mm [IQR]	50 [38–75]
Histology, n (%)	
Differentiated	72 (43.1)
Undifferentiated	92 (55.1)
Others	3 (1.8)
Pathological T factor, n (%)	
T1	16 (9.6)
T2	29 (17.4)
T3	55 (32.9)
T4	67 (40.1)
Pathological N factor, n (%)	
N0	78 (46.7)
N1	34 (20.4)
N2	26 (15.6)
N3	29 (17.4)
Pathological stage, n (%)	
I	39 (23.4)
II	63 (37.7)
III	61 (36.5)
IV	4 (2.4)
DLNM, n (%)	
Yes	8 (4.8)
No	159 (95.2)
Pathological tumor invasion to the M region, n (%)	
Yes	41 (24.6)
No	126 (75.4)

Abbreviations: DLNM, distal lymph node (stations No. 4d, 5, and 6) metastasis; IQR, interquartile range; M region, middle third of the stomach.

### 4.2 Rate of LN metastasis and therapeutic value index of LN dissection for each station

Table 3 shows the LN metastasis rate, 5‐y OS, and the therapeutic value index for dissection of each LN station. In patients with cT2 lesions, the metastatic rate and the therapeutic index of LN were both zero at stations No. 4d, 5, and 6. On the other hand, the LN metastasis rate was zero only at station No. 6 of the three stations examined patients with cT3/T4 tumors. Among the regional stations examined, however, the therapeutic indices of LN dissection at stations No. 4d and 5 were extremely low, at 1.0 and 1.4, respectively. The therapeutic index for dissection of LNs at station No. 12a was also zero in all patients.

**TABLE 3:** Metastatic ratio and therapeutic value index for dissection of LNs at each station

Station of LN	No. of metastatic LNs/retrieved LNs	Metastatic ratio (%)	5‐y overall survival (%)	Therapeutic index
(a) cT2 lesions				
1	9/65	13.8	44.4	6.2
2	5/58	8.6	80.0	6.9
3	19/65	29.2	78.9	23.1
4sa	3/58	5.2	66.7	3.4
4sb	3/61	4.9	33.3	1.6
4d	0/65	0.0	0.0	0.0
5	0/47	0.0	0.0	0.0
6	0/65	0.0	0.0	0.0
7	3/64	4.7	33.3	1.6
8a	1/65	1.5	0.0	0.0
9	2/62	3.2	50.0	1.6
10	2/19	10.5	50.0	5.3
11p	5/65	7.7	40.0	3.1
11d	2/26	7.7	0.0	0.0
12a	0/25	0.0	0.0	0.0

(b) cT3‐4 lesions				
1	25/101	24.8	40.0	9.9
2	20/96	20.8	45.0	9.4
3	46/102	45.1	54.3	24.5
4sa	10/87	11.5	30.0	3.4
4sb	6/97	6.2	16.7	1.0
4d	6/102	5.9	16.7	1.0
5	1/73	1.4	100.0	1.4
6	1/101	1.0	0.0	0.0
7	23/102	22.5	56.5	12.7
8a	3/102	2.9	33.3	1.0
9	8/97	8.2	50.0	4.1
10	10/70	14.3	20.0	2.9
11p	16/95	16.8	31.3	5.3
11d	6/60	10.0	16.7	1.7
12a	0/50	0.0	0.0	0.0

### 4.3 Patients with DLNM

Table 4 shows the clinicopathological and demographic characteristics of eight patients with DLNM. All primary lesions extended to the gastric body, and all had a depth of cT3 or cT4. All but one of the seven patients were diagnosed at far advanced stages of disease, pStage III or IV, with extensive LN metastasis.

**TABLE 4:** Characteristics of patients with DLNM

No.	Gender	Age, years	Distal border of tumor	Clinical macroscopic type	Preoperative tumor size, mm	cT factor	Histology	pT factor	pN factor	pStage
1	Female	67	Gastric body	Type 3	100	cT4a	Undiff.	pT4a	pN3b	IIIC
2	Female	61	Gastric body	Type 3	55	cT3	Undiff.	pT4a	pN3a	IVA
3	Male	71	Gastric body	Type 3	60	cT4a	Undiff.	pT4a	pN3b	IIIC
4	Male	79	Gastric body	Type 3	60	cT4b	Undiff.	pT4b	pN3b	IVA
5	Male	59	Gastric body	Type 3	40	cT3	Diff.	pT4a	pN3a	IIIC
6	Male	81	Gastric body	Type 3	60	cT4a	Diff.	pT4a	pN3a	IIIC
7	Male	73	Gastric body	Type 1	35	cT3	Diff.	pT1b	pN1	IB
8	Female	48	Gastric body	Type 3	50	cT4a	Undiff.	pT4a	pN3a	IIIC

Abbreviations: DLNM, distal lymph node (stations No. 4d, 5, and 6) metastasis; Undiff., undifferentiated type.

### 4.4 Patients with pathological tumor invasion to M region

Table 5 compares the clinicopathological factors between patients with and without pathological tumor invasion to the M region. The proportions of females and patients with cT4 lesions were significantly higher among those with than those without pathological invasion to the M region. Preoperative and pathological tumor sizes were significantly greater in patients with than in those without pathological invasion to the M region. In addition, none of the tumors limited to the cardia and/or the fornix based on preoperative endoscopy extended to the M region in the pathological specimen.

**TABLE 5:** Comparison between patients with and without pathological tumor invasion to the M region

	No pathological invasion to M region	Pathological invasion to M region	*P* value
	(n = 126)	(n = 41)	
Sex, n (%)			
Male	93 (73.8)	23 (56.1)	.032
Female	33 (26.2)	18 (43.9)	
Age, years [IQR]	67 [60‐73]	63 [54‐75]	.142
Tumor location, n (%)			
U	116 (92.1)	35 (85.4)	.205
UE	10 (7.9)	6 (14.6)	
Location of distal tumor border by endoscopy, n (%)			
Cardia or fornix	12 (9.5)	0 (0)	.040
Gastric body	114 (90.5)	41 (100)	
Clinical macroscopic type, n (%)			
0	25 (19.8)	10 (24.4)	.444
1	13 (10.3)	1 (2.4)	
2	29 (23.0)	10 (24.4)	
3	56 (44.4)	20 (48.8)	
5	3 (2.4)	0 (0)	
Preoperative tumor size, mm [IQR]	40 [30‐50]	50 [40‐60]	<.001
Clinical T factor, n (%)			
T2	56 (44.4)	9 (22.0)	<.001
T3	34 (27.0)	7 (17.1)	
T4	26 (28.6)	25 (61.0)	
Histology, n (%)			
Differentiated	57 (45.2)	15 (36.6)	.605
Undifferentiated	67 (53.2)	25 (61.0)	
Others	2 (1.6)	1 (2.4)	
Pathological tumor size, mm [IQR]	48 [35‐62]	80 [52‐95]	<.001
Pathological T factor, n (%)			
T1	15 (11.9)	1 (2.4)	.030
T2	24 (19.1)	5 (12.2)	
T3	44 (34.9)	11 (26.8)	
T4	43 (34.1)	24 (58.5)	
Pathological stage, n (%)			
I	34 (27.0)	5 (12.2)	.124
II	48 (38.1)	15 (36.6)	
III	42 (33.3)	19 (46.3)	
IV	2 (1.6)	2 (4.9)	

Abbreviations: IQR, interquartile range; M region, middle third of the stomach.

## 5 DISCUSSION

We evaluated the pathological status of regional LNs, the therapeutic index for each nodal station, and the pathological tumor location in patients undergoing TG for cT2–4 upper third gastric cancer, to explore the possibility of applying PG for these lesions. The following findings were obtained in the present study. Among the regional LNs examined, the therapeutic indices for dissection of LNs at stations No. 4, 5, 6, and 12a were zero or extremely low. Moreover, no DLNM was detected in cT2 lesions or cT3/T4 lesions located within the cardia/fornix. In addition, the lesions located within the cardia/fornix by preoperative endoscopy did not extend to the M region in the pathological specimen. Therefore, PG without No. 12a dissection might be indicated for lesions within the cardia/fornix, considering the oncological aspects of DLNM and the risk of cancer‐positivity in the distal margin.

Several studies have focused on the frequency of DLNM in locally advanced cancers in the upper third of the stomach, and all obtained similar results.^10^, ^11^, ^14^ In particular, Yura et al suggested that the frequency of DLNM in pT2/T3 lesions in the upper region was extremely low, and that no therapeutic effect was obtained by dissecting these LNs.^11^ However, patients were not studied according to clinical T factors, but instead only according to pathological T factors in all of the previous investigations. Given that surgical treatment is determined based on the preoperative diagnosis including the clinical T factor and that clinicopathological discrepancies among T categories are possible, the results obtained from the present study, which included taking clinical T factors into account, appear to be reliable.

Moreover, tumor locations were further divided into three regions based on preoperative endoscopic findings, revealing that DLNM is unlikely to develop in locally advanced lesions, even with serosal invasion, when they are located above the gastric body. Although greater tumor diameter is considered to carry a risk of deeper wall invasion,^15^, ^16^, ^17^ PG might be acceptable for such lesions because a sufficient distal margin can be maintained. In addition, given that all patients with DLNM were pathologically diagnosed as having far advanced disease stages, ie, pStage IIIC or IV, except in one case, surgical treatment alone would not have achieved satisfactory long‐term survival. Thus, patients with DLNM require perioperative multidisciplinary treatments.

As specified in the current guidelines,^6^ lymph node dissection with D1 or D1 plus can be applied in PG for early lesions, while the optimal extent of lymphadenectomy in PG for advanced stage cancer remains unknown. Although LNs at the distal stations cannot be removed because blood flow to the remnant stomach must be preserved, it is possible to dissect LNs at stations No. 10, 11d, and 12a, which are not included among the LNs defined as being suitable for PG with D1 or D1 plus. Based on several reports indicating that No. 12a LN dissection has no therapeutic effect, which is consistent with the results of the present study,^10^, ^11^, ^14^ it may be reasonable to omit lymphadenectomy of station No. 12a when performing PG for advanced lesions. On the other hand, the frequency of No. 10 LN metastasis in proximal advanced gastric cancer is not low, reportedly being 10.7%–16.5%,^18^, ^19^, ^20^ which is similar to the result obtained in this study. Splenectomy for No. 10 LN dissection should be determined according to the results of a randomized controlled trial.^21^

Insufficient volume of the remnant stomach after PG is reportedly associated with deterioration of postoperative quality of life and skeletal muscle loss.^22^, ^23^ In practice, the aim is generally to preserve more than 2/3 of the preoperative gastric volume in performing PG for early lesions.^7^, ^24^ Even when applying PG to advanced tumors, it is apparently essential that at least half of the distal stomach, as recommended in the guidelines, be preserved.^6^ Large tumors and pathologically advanced T stage are reportedly risk factors for a positive resection margin in gastric cancer surgery, similar to the characteristics of patients with pathological tumor invasion to the M region in this study.^25^, ^26^, ^27^ However, this study suggested that more than half of the stomach might be preserved even with locally advanced gastric cancer, provided that the lesion is located within the cardia and/or the fornix.

This study has several limitations. First, this was a retrospective study with a small sample size conducted at a single institution. Moreover, we were not able to perform a multivariate analysis because there were too few patients with DLNM in this cohort. The most recent cases could not be included in the present study due to this being a 5‐y survival analysis. Second, the therapeutic effect of dissecting LNs at station No. 11d remains unclear due to the insufficient number of cases. In addition, whether dissection of No. 3b LNs has any clinical benefit could not be evaluated because not all of the No. 3 LNs were recorded separately, ie, as No. 3a or 3b LNs, in our database. This is because the nodes at station No. 3 were divided into 3a and 3b LNs according to the revision of the 14th edition of the Japanese Classification of Gastric Carcinoma in 2010.^13^ When performing PG for locally advanced lesions, complete dissection of LNs at station No. 3 including the No. 3b might be essential, along with not leaving a significant volume of the remnant stomach due to the necessity of securing an appropriate margin. The optimal extent of LN dissection at station No. 3 is a topic for future study. Third, functions of the remnant stomach such as peristalsis and retention after PG for locally advanced cancer were not evaluated. A multicenter study with a large sample size is required to clarify these issues and overcome the limitations of this study.

In conclusion, for locally advanced gastric cancer in the upper third of the stomach, PG without No. 12a dissection might be acceptable for lesions located within the cardia and/or the fornix, given that neither DLNM nor a distal cancer‐positive margin was detected in these cases.

## DISCLOSURE

The protocol for this research project was approved by the Institutional Review Board of the Cancer Institute Hospital and conforms to the provisions of the Declaration of Helsinki. Approval No. 2017‐1187. Informed consent was obtained from all of the subjects.

Conflict of Interest: The authors have no conflicts of interest regarding this article to declare.
